# Chitosan Sponges Are Associated With Higher Rates of Wound Complications Compared to Calcium Sulfate Beads

**DOI:** 10.7759/cureus.38490

**Published:** 2023-05-03

**Authors:** Kelsey McKee, Joseph Easton, Brian Mullis, Ivan Hadad

**Affiliations:** 1 Plastic Surgery, Indiana University School of Medicine, Indianapolis, USA; 2 Plastic Surgery, University of Cincinnati Health, Cincinnati, USA; 3 Orthopaedic Surgery, Indiana University Health Methodist Hospital, Indiana University School of Medicine, Indianapolis, USA; 4 Plastic and Reconstructive Surgery, Indiana University School of Medicine, Indianapolis, USA

**Keywords:** absorbable antibiotics, sentrex, osteoset, chitosan sponge, calcium sulfate beads, implant infection, wound infection, unanticipated surgery, wound dehiscence

## Abstract

Background

In this study, we aimed to determine if there is a difference in the rates of wound dehiscence, delayed union, nonunion, and unanticipated surgery after the use of bioabsorbable local antibiotic-delivery systems (LADS), specifically comparing antibiotic-impregnated calcium sulfate pellets (Osteoset-T, Wright Medical Technology Inc., Arlington, TN, USA, hereafter referred to as beads) and chitosan sponge (Sentrex BioSponge, Bionova Medical, Germantown, TN, USA, hereafter referred to as sponges) in the management of acute and chronic extremity wounds.

Methodology

We conducted a retrospective comparative cohort study in the setting of a level 1 trauma center. All patients who received either beads or sponges as an adjunct to surgical debridement from January 2010 to December 2017 were included, and 136 patients met the inclusion criteria. The intervention studied was extremity wounds that were treated with bioabsorbable LADS, either beads or sponges. The main outcome measurement was wound dehiscence and the need for unanticipated surgery.

Results

Of the 136 patients in the study cohort, 78% (106/136) were treated with beads, and 22% (30/136) were treated with sponges. Of the 136 patients, 50 (37%) experienced wound dehiscence, and 49 patients required unanticipated surgery. Overall, 62% (31/50) of patients with wound dehiscence and 67.4% (33/49) of patients requiring unanticipated surgery were seen in the bead cohort (p = 0.0001 and 0.025, respectively). However, in multivariable analyses, we found that the odds of having wound dehiscence and undergoing unanticipated surgery were, respectively, 4.9 (p = 0.001) and 2.8 (p = 0.021) times more likely to occur in the sponge than in the bead group.

Conclusions

Sentrex sponges appear to be associated with higher rates of wound dehiscence and the need for unanticipated surgery compared to Osteoset beads.

## Introduction

Local antibiotic-delivery systems (LADS) are an attractive option for the prevention and treatment of extremity wound infections and osteomyelitis. The use of various LADS has been described in settings of acute open extremity trauma [1,2], treatment of osteomyelitis [3-5], and treatment of chronic nontraumatic extremity wounds, such as diabetic wounds [6,7]. However, there is a paucity of evidence comparing different types of LADS in the management of various extremity wounds.

The purpose of our study was to compare two different antibiotic-impregnated bioabsorbable LADS, namely, calcium sulfate beads (hereafter referred to as Osteoset beads) and chitosan sponges (hereafter referred to as Sentrex sponges), as a treatment adjunct for acute and chronic extremity wounds. We specifically focused on the outcomes relating to wound and bone healing, including wound dehiscence, nonunion, malunion, and the need for unanticipated subsequent surgical interventions. Our null hypothesis was there was no difference in either wound dehiscence or unanticipated surgery between the Osteoset bead and Sentrex sponge cohorts.

## Materials and methods

Following institutional review board approval, a retrospective review was performed of all patients at a single institution who were treated for traumatic orthopedic wound infections and/or osteomyelitis using beads or sponges as an adjunct to surgical debridement from January 2010 to December 2017. Patients were assigned to the bead or sponge cohort at the distinction of the primary orthopedic surgeon in the manner of a case-control study. This assignment pattern was approved by the institutional review board for the purposes of this retrospective study. Demographic data collected via electronic medical record (EMR) included patient age and gender; type of fracture or wound; abuse of tobacco or alcohol, history of peripheral vascular disease (PVD), diabetes mellitus (DM), or renal disease; date of initial surgery; and postoperative complications including the need for subsequent unanticipated surgical intervention, wound dehiscence, delayed bone union, nonunion, and the need for subsequent related surgical procedures for definitive soft-tissue coverage.

Inclusion criteria consisted of all patients aged 18 years or older treated with surgical debridement and implantation of either beads or sponges. Diagnoses included acute open extremity fractures, wound infections associated with musculoskeletal injury, and suspected or confirmed osteomyelitis, both acute and chronic. Patients were excluded if younger than 18 years old at the time of initial intervention, if they did not receive surgical treatment, or if they were not treated with beads or sponges. A total of 136 cases were included in the study. Patients were followed via EMR until they received definitive surgery for soft-tissue coverage (amputation, skin graft, local or pedicled flap, free flap) or were discharged from the clinic. In the case that a patient received soft-tissue coverage surgery and then developed subsequent wound dehiscence or breakdown, they continued to be followed until their final definitive surgery.

Once a patient was deemed to meet the inclusion criteria, various patient and injury/wound information was compiled. Patient comorbidities evaluated included a history of PVD, DM, and chronic renal disease, as documented in the EMR. These disease processes were selected because they are known to have either a significant impact on wound healing or on the metabolism of various systemic antibiotics, which could alter a patient’s treatment plan or impact wound healing. Additional patient-related factors recorded included a history of tobacco or alcohol abuse. If diagnosed with an acute extremity fracture, they were classified as either upper or lower extremity. Open extremity fractures were graded according to the Gustilo and Anderson classification system [8]. Patients being treated for other wound types were classified only as the upper or lower extremity.

For treated extremity fractures, the development of delayed union or nonunion was assessed, as documented in both inpatient and outpatient EMR or on radiographic reports. Delayed union was defined as a fracture repair arrest of fewer than six months, and nonunion was defined as a fracture repair arrest of six months or more [9]. Documentation of surgical wound dehiscence was noted in a similar manner for all treated wound types. Previous clinical studies with bioabsorbable LADS have documented sterile serous drainage from wounds that often self-resolve. With this in mind, we did not consider the presence of simple serous drainage expressed from punctate openings (<5 mm) that resolved spontaneously and did not require prolonged local wound care, negative-pressure wound therapy, or a return to the operating room as dehiscence. Incision edge separations larger than punctate openings (>5 mm) that were managed with outpatient wound care, negative-pressure wound care, or return to the operating room were considered wound dehiscence.

Evaluated postoperative outcomes also included unexpected repeat admission within both 30 days and one year. The need for unanticipated surgery was noted within this time period, which included surgery for wound healing complications, implant infections or failure, and bone healing complications, such as delayed union or nonunion. Multiple repeated surgical debridements for wounds that were left open or temporarily treated with negative-pressure wound devices were considered “anticipated,” as these were presumed to need to return for definitive surgical closure. Similarly, if it was documented in the operative report that the patient would need to return for additional surgical debridement, the subsequent surgery was also classified as “anticipated.” For those procedures considered to be unanticipated, the surgical procedures were generally classified as primary closure, amputation, skin graft, pedicled flap (including muscle, myocutaneous, and fasciocutaneous), and free flap (including muscle, myocutaneous, and fasciocutaneous). This classification was only applied to the procedure that ultimately resulted in definitive wound closure.

The study cohort was evaluated in univariate analyses using mean (±SD) and percentages. In bivariate analysis, the t-test was used for continuous variables, and Pearson’s chi-square and Fisher’s exact tests were used as appropriate to examine if either of the two treatment groups was associated with patients’ demographic, medical history, wound dehiscence, and unanticipated surgery. Multivariable logistic regression models were used to examine the consecutive association of the treatment group with wound dehiscence and unanticipated surgery after adjusting for the effect of age, gender, history of diabetes, alcohol consumption, renal disease, and smoking behavior. Adjusted odds ratios (ORs) with 95% confidence intervals (CIs) were reported. All hypotheses were tested at a 0.05 level of significance, and the analyses were done using Stata/SE 14.2 (StataCorp. 2015. Stata Statistical Software: Release 14. College Station, TX, USA).

## Results

Of the 136 patients in the study cohort, the mean age was 44.4 (±14.1) years and 31.6% (43/136) were female. Regarding the history of comorbidities, 55.9% (76/136) were smokers, 25.7% (35/136) consumed alcohol, and 30.2% (41/136) had diabetes. Only 3.7% (5/136) had documented PVD, and 5.9% (8/136) had renal disease. In total, 106 (77.9%) patients were treated with beads, whereas the remaining 30 (22%) were treated with sponges (Table 1). Slightly more than one-third of the patients had reported wound dehiscence and unanticipated surgery (36.7% (50/136) and 36.0% (49/136), respectively).

**Table 1:** Characteristics of the study cohort in the sample and by treatment groups. PVD = peripheral vascular disease; DM = diabetes mellitus

Variables	Total = 136		Osteoset = 106		Sentrex = 30		P-value
	n	%	n	%	n	%	
Wound dehiscence							0.0001
No	86	63.24%	75	87.21%	11	12.79%	
Yes	50	36.76%	31	62.00%	19	38.00%	
Unanticipated Surgery							0.025
No	87	63.97%	73	83.91%	14	16.09%	
Yes	49	36.03%	33	67.35%	16	32.65%	
Age at surgery, mean (SD)	44.29 (14.07)		44.15 (14.20)		45.23 (13.82)		0.7114
Gender							0.046
Male	93	68.38%	68	73.12%	25	26.88%	
Female	43	31.62%	38	88.37%	5	11.63%	
Fracture: open							0.663
No	44	51.76%	35	79.55%	9	20.45%	
Yes	41	48.24%	31	75.61%	10	24.39%	
Fracture: grade							0.395
1	2	4.88%	1	50.00%	0	0.00%	
2	3	7.32%	2	66.67%	1	33.33%	
3	19	46.34%	12	63.16%	7	36.84%	
4	16	39.02%	14	87.50%	2	12.50%	
5	1	2.44%	1	100.00%	0	0.00%	
PVD							>0.999
No	131	96.32%	102	77.86%	29	22.14%	
Yes	5	3.68%	4	80.00%	1	20.00%	
DM							0.984
No	95	69.85%	74	77.89%	21	22.11%	
Yes	41	30.15%	32	78.05%	9	21.95%	
Renal disease							0.374
No	128	94.12%	101	78.91%	27	21.09%	
Yes	8	5.88%	5	62.50%	3	37.50%	
Alcohol							0.043
No	101	74.26%	83	82.18%	18	17.82%	
Yes	35	25.74%	23	65.71%	12	34.29%	
Smoking 10 packs							0.352
No	60	44.12%	49	81.67%	11	18.33%	
Yes	76	55.88%	57	75.00%	19	25.00%	

In the bivariate analysis, comparing between two treatment groups, it was found that 26.9% (n = 25/93) of the male patients were in the chitosan sponge group, whereas 11.6% (n = 5/43) of the female patients were in the calcium sulfate bead group (p = 0.046). The proportion of the documented history of alcohol consumption was observed to be higher in the bead group than in the sponge group (p = 0.043). There was no difference between the treatment groups regarding the age at surgery, fracture type, history of PVD, diabetes, renal disease, and smoking behavior. Overall, 62.0% (31/50) of the wound dehiscence cohort (p = 0.0001) and 67.4% (33/49) of the unexpected surgery (p = 0.025) cohort were seen in the patients receiving beads (Table 1).

In the multivariable analysis of wound dehiscence (Table 2), after controlling for the effect of age, gender, history of diabetes, alcohol consumption, renal disease, and smoking behavior, it was found that wound dehiscence was 4.88 times more likely to occur among those in the sponge group than those in the bead group (p = 0.001). Smoking was also independently associated with an increased likelihood of having wound dehiscence (adjusted OR = 4.3, 95% CI = 1.8-10.4, p = 0.001). Age at surgery, gender, history of having diabetes, renal disease, and alcohol consumption were not independent predictors of wound dehiscence (p > 0.05).

**Table 2:** Multivariable logistic regression of wound dehiscence and unanticipated surgery. OR = odds ratio; CI = confidence interval

Characteristics	Wound dehiscence		Unanticipated surgery	
	Adjusted OR (95% CI)	P-value	Adjusted OR (95% CI)	P-value
Treatment				
Osteoset	Reference			
Sentrex	4.89 (1.90, 12.58)	0.001	2.83 [1.17, 6.84]	0.021
Age at surgery	0.99 (0.96, 1.02)	0.367	1.01 [0.98, 1.04]	0.721
Gender				
Male	Reference			
Female	1.17 (0.49, 2.76)	0.726	0.85 (0.37, 1.92)	0.691
Diabetes				
No	Reference			
Yes	1.12 (0.42, 2.93)	0.825	1.46 (0.6, 3.56)	0.405
Alcohol				
No	Reference			
Yes	0.66 (0.25, 1.72)	0.39	0.6 (0.23, 1.57)	0.295
Renal disease				
No	Reference			
Yes	0.62 (0.11, 3.53)	0.593	0.33 (0.06, 1.93)	0.218
Smoking 10 packs				
No	Reference			
Yes	4.3 (1.79, 10.37)	0.001	1.36 (0.61, 3)	0.452

In the second multivariable analysis of unanticipated surgery (Table 2), it was found that unanticipated surgery was 2.8 times more likely in the sponge group compared to the bead group (adjusted OR = 0.35, 95% CI = 0.15-0.85, p = 0.021) after controlling for the effect of age, gender, history of diabetes, alcohol consumption, renal disease, and smoking behavior.

Multivariable penalized logistic regression was also performed for delayed union and nonunion to account for the sparsity of these outcomes. No significant difference between the bead and sponge groups was found.

**Figure 1:**
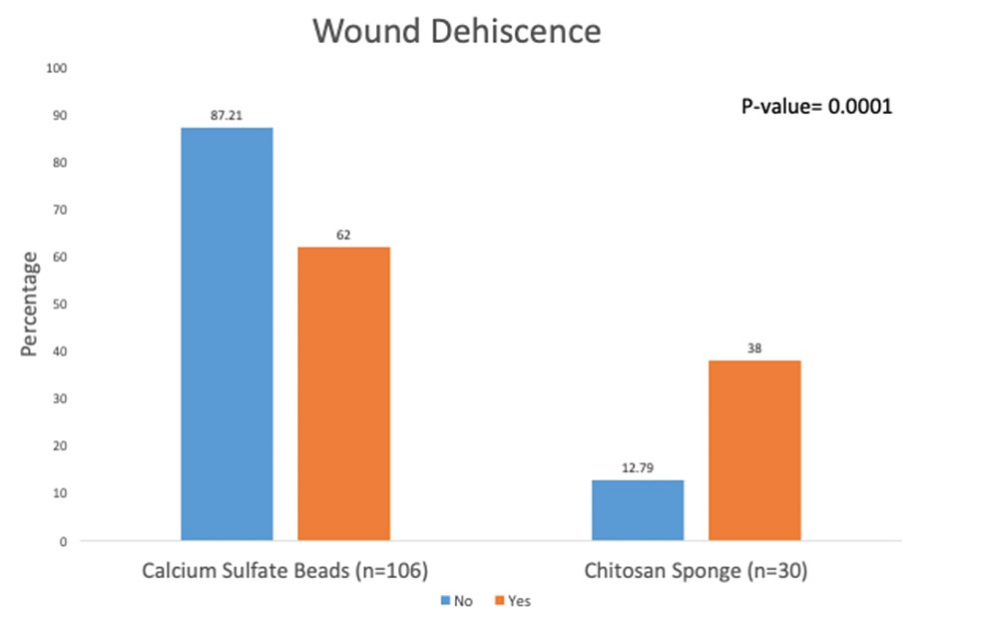
Wound dehiscence.

**Figure 2:**
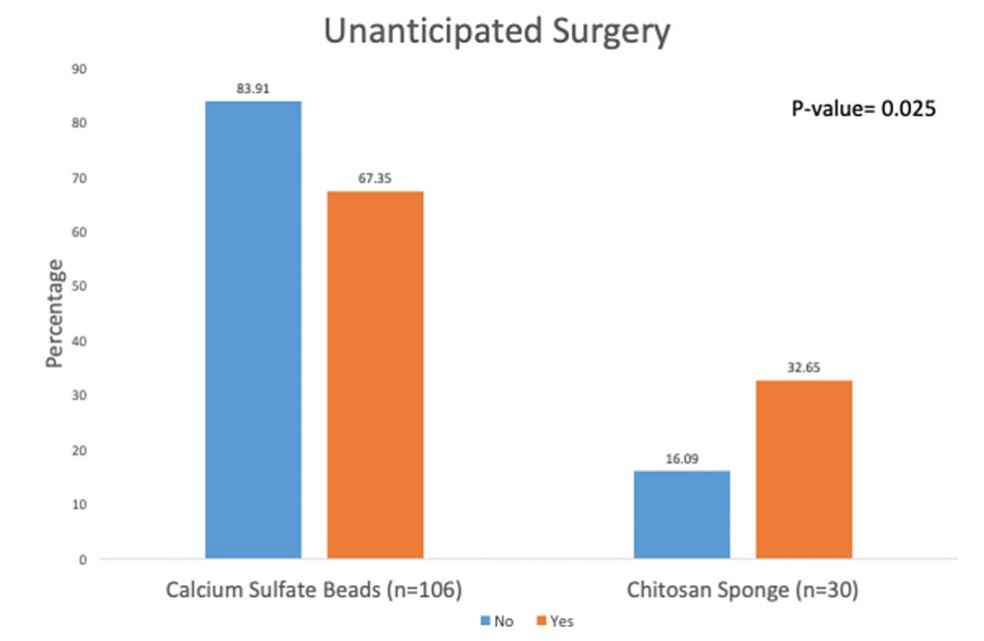
Unanticipated surgery.

Our multivariable analysis results demonstrate that sponges were associated with a higher rate of wound dehiscence (Figure 1) and unanticipated surgical intervention (Figure 2) than beads after controlling for patient comorbidities.

## Discussion

The use of LADS has been described in a wide variety of clinical scenarios, including the treatment of acute open fractures, subacute and chronic wounds, and osteomyelitis. Patients suffering from these various wound types are commonly encountered in the healthcare field, and the associated health and economic burden of these wounds have been well described. For example, diabetic foot ulcers alone cost Medicare $9-13 billion per year, with most of these costs driven by surgical procedures [9]. Similarly, traumatic extremity fractures are estimated to cost nearly $2 billion per year [3]. When infectious complications are present, this only adds to the healthcare burden due to repeat hospitalizations and revision surgeries. Furthermore, various studies have demonstrated that extremity injuries and wounds often lead to a loss of function and long-term disability [3,10-12]. This emphasizes the importance of investigating additional and improved treatment options when addressing complex extremity wounds.

The current standard treatment for these wounds with surgical debridement and systemic antibiotics may be insufficient due to poor antibiotic delivery to the wound itself, possibly related to tissue injury, vascular disease or injury, poor bone penetration, or bacterial biofilm formation. This has led to the development of a wide variety of LADS.

The application of antibiotics locally into the wound space itself is not a new concept. Polymethylmethacrylate (PMMA) use as an antibiotic carrier in orthopedic wounds was first described in Europe in the 1980s and was previously considered to be the gold standard [1]. However, it is not an ideal delivery system because it is not biodegradable, and therefore, would require additional surgery to remove it. If left in place, studies have shown that it can even foster the development of antibiotic-resistant bacteria and biofilm [1,3]. Due to these issues, various bioabsorbable delivery systems, including calcium sulfate and chitosan products, have been developed and studied.

Open traumatic extremity fractures are associated with high rates of patient morbidity and cost due in large part to subsequent infections and associated wound complications [2]. Osteomyelitis alone accounts for nine out of every 10,000 hospital admissions in America annually [13] and has traditionally been a difficult bacterial infection to treat. The current standard treatment for osteomyelitis is a combination of surgical debridement and irrigation with systemic antibiotics [14]. However, some wounds are not amenable to systemic antibiotic delivery due to traumatic injury of surrounding tissue, chronic or acute vasculature disruption, and poor antibiotic penetration of bone [2]. Additionally, the formation of biofilm by bacterial colonies can confer antibiotic resistance more than 1,000 times higher than normal therapeutic antibiotic concentrations [5]. These biofilm-forming bacteria are the cause of more than 65% of infections treated in the developed world [15]. This paradigm has led to innovations in local antibiotic administration as an adjunct for the treatment of infected traumatic wounds and osteomyelitis.

LADS can be advantageous in their ability to achieve high antibiotic concentrations locally while minimizing systemic toxicities [5,14]. Various LADS exist, including antibiotic-impregnated PMMA cement, calcium sulfate, and chitosan sponges, among others. However, there are few studies comparing LADS for the treatment of acute and chronic extremity wounds and osteomyelitis.

Calcium sulfate embedded with antibiotics has been well studied and used for several decades for the prevention and treatment of orthopedic wounds, although it should be noted this is an off-label use in the United States. It is an inorganic compound that dissolves fairly quickly, making it effective at delivering high local levels of antibiotics. As calcium sulfate dissolves, it creates an acidic environment, which is thought to cause local wound issues, often manifested as serous drainage [4,5,16]. This has been noted to occur anywhere from 4-51% [1,4,17], and is often reported as self-limiting and resolves as the calcium sulfate is resorbed [18-20]. However, it was noted in a study by Humm et al. [4] that three-quarters of their cases with wound drainage went on to have wound healing problems.

More recently, increasing use of chitosan sponges impregnated with antibiotics has been noted. Chitosan is an abundant polysaccharide made from shellfish, such as shrimp and crab [1,17]. It is known to have inherent wound-healing properties and can be altered to have predictable antibiotic elution rates. However, clinical studies of chitosan sponges as local antibiotic carriers remain limited. To date, there are no studies of clinical outcomes comparing LADS Osteoset-T calcium sulfate beads and Sentrex chitosan sponges for the treatment of extremity injuries and osteomyelitis.

Our study has several limitations. First, it is a retrospective study and thus subject to some bias relating to inaccurate or inconsistent reporting in the EMR. Second, the study is limited by the number of patients in the chitosan sponge cohort. This is due in part to the cessation of product use when it was clinically noted that more of these patients seemed to have an increased proportion of wound healing complications. Third, there might be residual confounding effects in the model as we were not able to account for some confounders, for example, PVD and open fracture type. However, in this data, we checked that renal disease, which was used as a confounder in the multivariable model, did not vary with the open type of fracture, and the confounding effect was not observed for PVD when renal disease was included in the model. Hence, to maintain parsimony of the model, we conclude that in this cohort, PVD and open fracture types did not affect our results in the multivariable analysis. Finally, though there are studies that highlight the effect of alcohol intake on wound dehiscence, our study was not able to detect this effect. This might be due to study power or there might be several individuals with less alcohol intake. Because we were not able to collect the amount of alcohol intake reliably from the patient chart review, future studies exploring the dose-response relationship between alcohol intake and wound dehiscence are recommended.

## Conclusions

Our study sought to compare the rates of wound dehiscence and the need for further unanticipated surgical intervention based on two different LADS. The results of our study suggest that chitosan sponges are associated with a higher wound complication rate and need for unanticipated surgery compared to antibiotic-impregnated calcium sulfate beads. Further higher level of evidence such as prospective randomized studies would be needed to confirm these results.
